# Vacuum-assisted closure therapy in ureteroileal anastomotic leakage after surgical therapy of bladder cancer

**DOI:** 10.1186/1477-7819-5-41

**Published:** 2007-04-12

**Authors:** Stefan Denzinger, Lars Luebke, Maximilian Burger, Sigurd Kessler, Wolf F Wieland, Wolfgang Otto

**Affiliations:** 1 Department of Urology, University of Regensburg, Regensburg, Germany; 2 Department of Surgery, Ludwig-Maximilian University of Munich, Munich, Germany

## Abstract

**Background:**

Vacuum-assisted closure (VAC) is an acknowledged method of treating wound healing disorders, but has been viewed as a contraindication in therapy of intraabdominal fistulas.

**Case presentation:**

We present the case of an 83-year old patient with ureteroileal anastomotic insufficiency following cystectomy and urinary diversion by Bricker ileal conduit due to urothelial bladder cancer. After developing an open abdomen on the 16^th ^postoperative day a leakage of the ureteroileal anastomosis appeared that cannot be managed by surgical means. To stopp the continued leakage we tried a modified VAC therapy with a silicon covered polyurethane foam under a suction of 125 mmHg. After 32 days with regularly changes of the VAC foam under general anesthesia the fistula resolved without further problems of ureteroileal leakage.

**Conclusion:**

We present the first report of VAC therapy successfully performed in urinary tract leakage after surgical treatment of bladder cancer. VAC therapy of such disorders requires greater care than of superficial application to avoid mechanical alterations of internal organs but opens new opportunities in cases without surgical alternatives.

## Background

In 1995 Morykwas and Argenta introduced vacuum-assisted closure (VAC) into the management of complex wound healing disorders [1]. Negative pressure is established in the wound area by applying suction through a fitted polyurethane foam secured by adhesive tape dressing. The negative pressure drains wound exudate continuously and reduces edema and bacterial load. Furthermore, granulation tissue formation and angioneogenesis are stimulated. These factors accelerate wound healing. The classical indications for VAC therapy are decubitus ulcers, leg ulcers, posttraumatic and postoperative wounds, mesh grafts, sternal wound infections and open abdomen. In the current literature the feasibility of VAC in intraabdominal fistulas is debated as mechanical alterations can cause intestinal damage [2]. The presence of malignant tissue is generally viewed as a contraindication for VAC, as it stimulates cell growth [1]. There are only three reports of VAC in urology [2-4]. No treatment of intraabdominal urinary leakage, a rare but severe event in urinary diversion [5], has been reported to date. We present the initial report of a successful treatment of an ureteroileal anastomotic insufficiency after cystectomy and Bricker ileal conduit urinary diversion due to bladder cancer by VAC therapy.

## Case presentation

**Figure 1:**
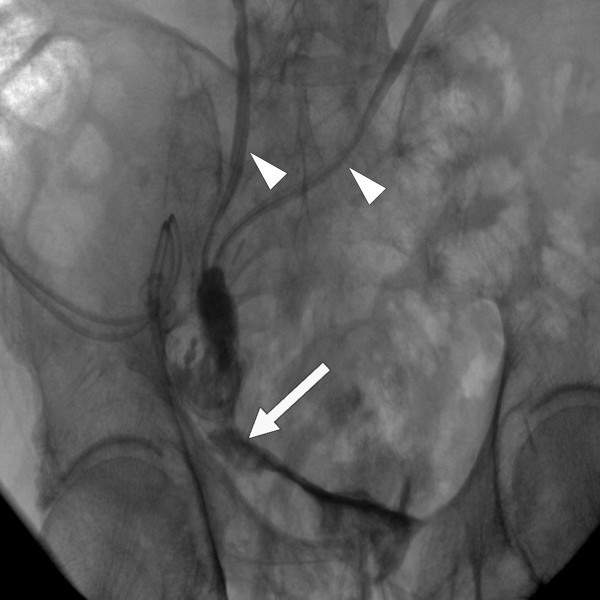
Anastomotic insufficiency (white arrow) in radiologic control. Small white arrows point at ureters.

**Figure 2:**
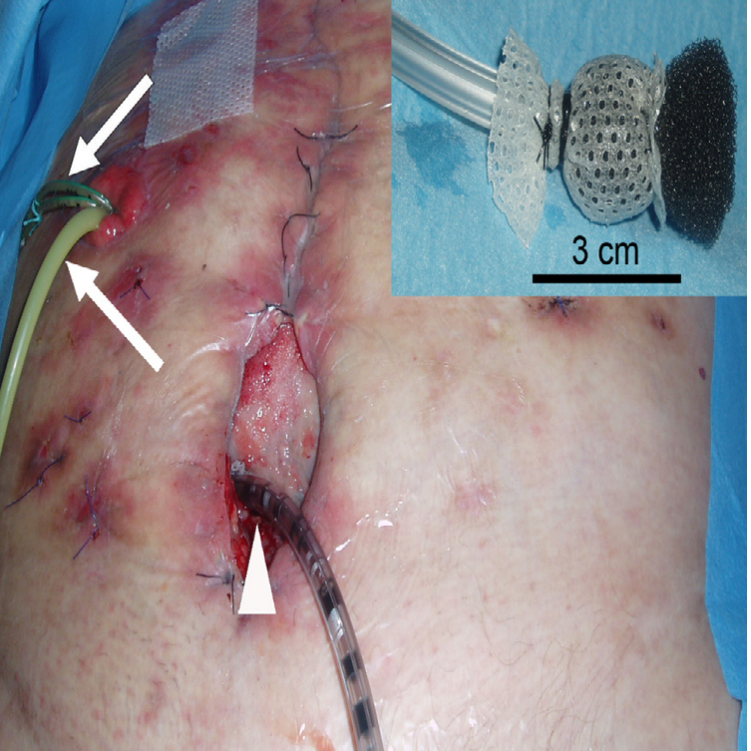
VAC foam covered by Mepithel^® ^wound around VAC tube (small picture, right corner). Tube placed (white arrow head) through abdominal wound and VAC dressing applied. Foley-catheter and ureteral catheters inserted through conduit (white arrows).

**Figure 3:**
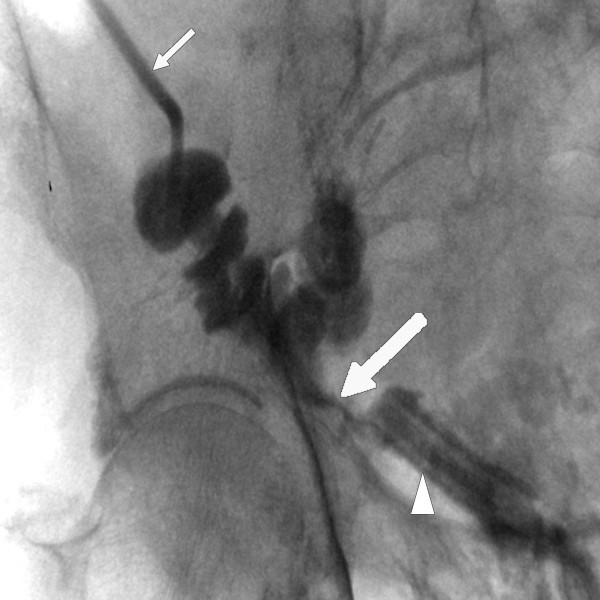
Positioning of VAC tube (white arrow head): radiologic guidance by instilling contrast agent through conduit. White arrow points at leakage of urine. Contrast agent applied through catheter in ileal conduit (small white arrow).

**Figure 4:**
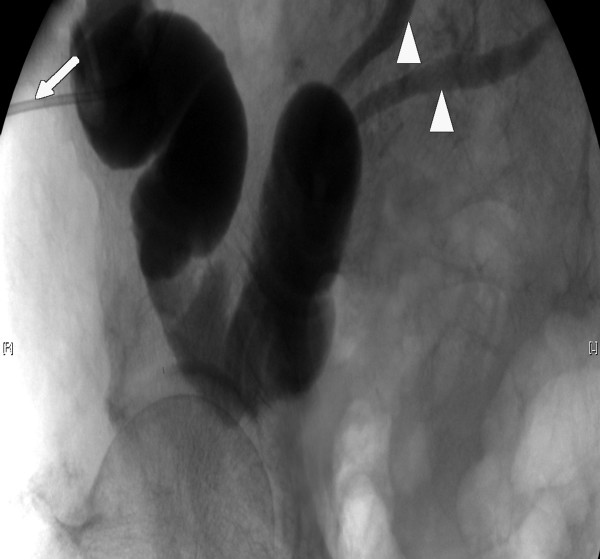
Resolved anastomotic insufficiency in radiologic control; contrast agent applied through catheter in ileal conduit (white arrow). Small white arrow heads point at ureters.

A 83-year old patient in good medical condition underwent radical cystectomy and urinary diversion by Bricker ileal conduit for pT3a, G3, pN0, R0 urothelial bladder cancer. On the 3^rd ^postoperative day, wound drains showed fecal secretion and relaparotomy was undertaken. Intraoperatively, massive adhesions and ileoileal anastomotic insufficiency were found. Extensive lavage of the abdomen was performed, the former ileoileal anastomosis resected and reconstructed. Severe wound healing disorder occurred resulting in an open abdomen. A further relaparotomy and extensive lavage were performed on the 9^th ^postoperative day; despite massive adhesions full overview of the surgical site could be obtained. There was no macroscopic sign of anastomotic leakage at this point. As primary wound closure could not be achieved, open wound care was initiated consisting of wet saline dressings changed every two days following minor abdominal lavage. On the 16^th ^postoperative day, leakage of urine was suspected due to increased secretion of the wound and confirmed by radiographic imaging. Relaparotomy was mandated, ureteroileal anastomosis reconstructed and two nephrostomies placed. Persistent exudation led to the assumption of continued anastomotic insufficiency, which was confirmed by flexible endoscopy and radiologic control (figure 1). The leakage probably due to the patient's advanced age accompanied by therefore deteriorated wound healing involved half the circumference of the ureteroileal anastomosis. A novel relaparotomy was initiated but futile due to pronounced adhesions. For lack of surgical strategies modified intraabdominal VAC therapy was employed to close the leakage. A piece of polyurethane foam was covered by Mepithel^®^, a silicon wound dressing, to avoid injury of the intestines and wound around a tube (figure 2). The tube was inserted through the abdominal wound and positioned at the leakage under radiologic control by instilling contrast agent through the conduit (figure 3). All changes of the device (duration about 40 minutes) with inspection of the intraabdominal situation by flexible endoscopy to rule out injuries of the intestines were performed by the same surgeon with the patient under general anesthesia in a room with the possibility of radiologic control. VAC dressing was applied as previously described and the tube led out of the abdomen through an polyurethan foam padding the open abdomen was connected to the VAC unit. After fixing the foam to the skin with special adhesive tapes suction was adjusted to 125 mmHg. While changing of the device was projected every three days, two extracurricular changes had to be performed due to increased mucosal output of the conduit clogging the foam. Within 32 days of VAC therapy the intraabdominal fistula resolved as demonstrated by radiological control (figure 4). For the remaining wound defect of the abdomen conventional wound care and secondary closure after 15 days were employed. Final endoscopic and radiologic controls of the conduit showed gradual mucosal coverage in the area of the anastomosis. The patient recovered well and was admitted to a rehabilitation unit. After a follow-up period of 13 months no further anastomotic insufficiency was noted.

## Discussion

Vacuum-assisted closure (VAC) is a recognized tool in the management of complex wound healing disorders expanding the spectrum of surgical strategies. While enteral leakage has been viewed as a contraindication for VAC, previous singular reports of successful treatment of intestinal anastomotic insufficiency [6-10] disprove this notion. While no application in urinary leakage has been reported to date, even in complex insufficiencies involving mucus and urine, intraabdominal granulation can be obtained. Suction by the fitted polyurethane foam lead to an absorption of the leaking urine and intestinal mucus that can disturb wound healing in the anastomotic area like in our case. In addition granulation tissue formation and angioneogenesis that means no problem after complete tumor resection are stimulated further accelerating wound healing. Whereas the application of VAC in superficial i.e. cutaneous wound defects is a largely standardized procedure and broadly applied, intraabdominal use requires great care as intestinal abrasion causing novel leakage may occur due to mechanical alterations. But Bricker ileal conduit today being the most uncomplicated urinary diversion system in treatment after radical cystectomy and no chance to close the urinary leakage by surgical means we had to try VAC therapy. Accurate inspection of the surrounding area, especially of adjacent organs such as the intestines, is necessary. Provided careful surveillance, even complex intraabdominal enteral leakages involving the urinary tract can be closed by local administration of VAC in a transperitoneal approach. Remaining abdominal wounds will heal in the absence of urine leakage impairing wound healing.

## Conclusion

We present the first report of vacuum-assisted closure therapy successfully performed in urinary tract leakage after surgical treatment of bladder cancer. Appreciated in superficial wound healing disorders VAC even may be considered in the face of lacking surgical alternatives for the closure of complex anastomotic insufficiencies.

## Competing interests

The author(s) declare that they have no competing interests.

## Authors' contributions

**SD **and **WO **drafted the manuscript, **LL **treated the patient and provided the Case report, **MB **and **SK **helped to draft the manuscript. **WFW **supervised treatment and draft of the manuscript.

## References

[B1] Argenta LC, Morykwas MJ (1997). Vacuum-assisted closure: a new method for wound control and treatment: clinical experience. Ann Plast Surg.

[B2] Whelan C, Stewart J, Schwartz BF (2005). Mechanics of wound healing and importance of Vacuum Assisted Closure in urology. J Urol.

[B3] Rosser C, Morykwas M (2000). A new technique to manage perineal wounds. Infect Urol.

[B4] Denzinger S, Luebke L, Roessler W, Wieland WF, Kessler S, Burger M (2006). Vacuum-assisted closure vs. conventional wound care in the treatment of wound failures following inguinal lymphadenectomy for penile cancer: a retrospective study. Eur Urol.

[B5] Wiesner C, Thuroff JW (2004). Techniques for uretero-intestinal reimplantation. Curr Opin Urol.

[B6] Gracias VH, Braslow B, Johnson J, Pryor J, Gupta R, Reilly P, Schwab CW (2002). Abdominal compartment syndrome in the open abdomen. Arch Surg.

[B7] Navsaria PH, Bunting M, Omoshoro-Jones J, Nicol AJ, Kahn D (2003). Temporary closure of open abdominal wounds by the modified sandwich-vacuum pack technique. Br J Surg.

[B8] Cro C, George KJ, Donnelly J, Irwin ST, Gardiner KR (2002). Vacuum assisted closure system in the management of enterocutaneous fistulae. Postgrad Med J.

[B9] Medeiros AC, Aires-Neto T, Marchini JS, Brandão-Neto J, Valença DM, Egito EST (2004). Treatment of Postoperative Enterocutaneous Fistulas by High-Pressure Vacuum with a Normal Oral Diet. Dig Surg.

[B10] Erdmann D, Drye C, Heller L, Wong MS, Levin SL (2001). Abdominal wall defect and enterocutaneous fistula treatment with the Vacuum-Assisted Closure (V.A.C.) system. Plast Reconstr Surg.

